# 

**Published:** 1891-04

**Authors:** 


### Societies—Illinois State Meeting.

To THE EDITOR OF THE DENTAL REGISTER:

*Dear Sir:*—The following programme is arranged for the Illinois State Dental Society to meet at Bloomington, May 12, 13, 14 and 15, 1891.

1. Annual address by the president, Dr. Truman W. Brophy, of Chicago.

2. Report of committee on dental science and literature, by Dr. J. D. Moody, of Mendota, Chairman.

3. Report of the committee on dental art and mechanism by Dr. W. B. Ames, of Chicago, Chairman.

4. How to deal with the condemned pulp by Dr. J. G. Dickson, of Carmi. Discussion to be opened by Dr. J. G. Reid, of Chicago.

5. The preparation of teeth for filling, by Dr. Edmund Noyes, of Chicago. Discussion to be opened by Dr. George H. Cushing, of Chicago.

6. Prosthetic dentistry, by Dr. W. T. Magill, of Rock Island. Discussion to be opened by Dr. E. C. Stone, of Galesburg.

7. Third period in the history of dentistry, continued with biographical notes, by Dr. John J. R. Patrick, of Belleville.

8. Experimental studies on the action of diffusible medicinal agents in living and pulpless teeth, by Dr. A. W. Harlan, of Chicago. Discussion to be opened by Dr. P. J. Kester, Chicago.

9. Architecture of the first upper molar, by Dr. Alton H. Thompson, of Topeka, Kansas. Discussion to be opened by Dr. A. B. Freeman, of Chicago.

10. A lantern view of the pulp chambers and canals, showing typical forms and variations, by Dr. D. M. Cattell, of Chicago. Discussion to be opened by Dr. G. V. Black, Jacksonville.

11. Efficiency and simplicity in regulating appliances, by Dr. E. H. Angle, of Minneapolis, Minn. Illustrated by stereopticon. Discussion to be opened by Dr. C. S. Case, Jackson, Mich.

12. Low fusing continuous gum body, by Dr. George Cunningham, of Cambridge, England. Discussion opened by Dr. W. B. Ames, Chicago.

13. Report of Supervisor of Clinics, by Dr. Louis Ottofy, of Chicago, Chairman. With discussion.

Dr. W. O. Kulp, Davenport, Clinics, Iowa. Art Technique.

Dr. I. P. Wilson, Burlington, Iowa. Root-filling.

Dr. R. H. Mace, Belleville, Ills. Filling at the cervical border using extension arm cervix clamp, also, a new suspension removable bridge.

Dr. W. B. Ames will demonstrate Dr. Cunningham's low fusing body for continuous gum.

Dr. Edgar Palmer, of La Crosse, Wis. Administration of narcotic vapors, using valved inhalers.

Dr. A. O. Hunt, Iowa City, Iowa. A new bridge.

Dr. E. E. Hughes, Des Moines, Iowa. Gold inlay.

Dr. Geo. H. Slyfield, Waukegan, Ills. Contour gold filling.

Dr. K. B. Davis, Springfield, Ills. Artificial crown.

Dr. S. F. Duncan, Joliet, Ills. Method of striking up crown tips.

It is particularly requested that those having pathological specimens, peculiar cases, models, new appliances and methods, will bring them to the meeting.

All practitioners of Illinois (including non-members) and of other States, are cordially invited to attend. They are especially urged to be present at the opening day and to remain through to the last session.

The usual reduction in hotel rates and railroad fares will be allowed. J. W. WASSALL, Chairman Executive Committee.

### Northern Ohio Dental Association.

The forty-second annual meeting of this body will be held at Oberlin, O., in the Y. M. C. A. building, Tuesday, May 12th, 1891, at 10 o'clock A.M. continuing three days. A very interesting program has been prepared, embracing the following subjects, viz.:

"Development of the Teeth," paper by Dr. W. H. Whitslar, of Youngstown, discussion opened by Dr. A. J. Dowds, of Canton; second, "The Recurrence of Decay in Teeth," paper by Dr. J. G. Templeton, of Pittsburg, Pa.; third, paper by Dr. W. H. Atkinson, New York, subject, "Hind Sight," discussion opened by Drs. C. R. Butler, Cleveland, and E. J. Waye, Sandusky; fourth, "Sanitary Condition of the Mouth, and How Best to Maintain It," paper by Dr. J. F. Dougherty, of Canton, discussion opened by Dr. W. T. Jackman, of Cleveland, and Dr. J. H. Wible, of Canton; there will also be presented a number of voluntary papers, incidents of office practice, and several clinics, among which are the following: "A Tin Filling with Gold Facing," by Dr. S. B. Dewey, Cleveland; second, "Electricity Supplemented with a paper," by Dr. Frank Creger, Fremont, O.; third, "Melting an Ingot of Amalgam," followed by a talk on the same, by Dr. J. F. Siddall, Oberlin, Ohio.

This promises to be an unusually interesting meeting and we hope that all the dentists who can will make arrangements to attend and carry with them any new appliances, devices, models of interesting cases, or report of some case in practice.

Any desired information may be had by addressing Dr. Henry Barnes, 106 Euclid Av., Cleveland, O.

### SOCIETIES.

### American Medical Association, Section of Oral and Dental Surgery.

The forty-second session of the American Medical Association will be held in Washington, D. C., on Tuesday, Wednesday, Thursday and Friday, May 5th, 6th, 7th and 8th, commencing on Tuesday at 11 o'clock, A. M.

The following is a list of Essayests (with subjects), who have promised to prepare papers for the section of Oral and Dental Surgery.

1.—Address of the Chairman of Section, Dr. Eugene S. Talbot.

2.—Adenoid Growth, Dr. W. H. Atkinson.

3.—Treatment of fractures of the Maxillas, Dr. Wm. Carr.

4.—Genesis of Contour Fillings, Illustrated, Dr. George S. Allan.

5.—The teeth of Invertebrate Animals, Dr. A. H. Thompson.

6.—A Study in Comparative Dental Anatomy, Dr. Wm. H. Potter.

7.—Rheumatic and Gouty Diathesis as manifested in diseases of the Peridental Membrane, Dr. John S. Marshall.

8.—Dental Infirmery Patients—The Use and Abuse of Dental Charity, Dr. Richard Grady.

9.—Growth of the Cementum, Dr. R. R. Andrews.

10.—Remarks on Incipiant Necrosis and Caries, Dr. J. L. Williams.

11.—Choice of Therapeutic Filling Materials, Dr. W. W. Allport.

12.—Care of the Teeth, Dr. J. Taft.

13.—Thorough Dentistry vs Partial Dental Surgery, Dr. J. Y. Crawford.

14.— Dr. Thos. Fillisbrown.

Other members who desire to read papers before this section should, as required by the By-Laws, forward the paper, or its *title* and *length*, to the Chairman, Dr. Eugene S. Talbot, 125 State Street, Chicago, Ills., one month before the Meeting.

HENRY W. MORGAN,

Secretary.

Nashville, Feb. 23d, '91.

---

THE next Meeting of the National Association of Dental Examiners will be held at Saratoga, probably on Saturday, August 1st, 1891. It has not seemed practicable to arrange an earlier time for the meeting. It is to be hoped that all of the Boards in the country will be represented. T.

### CONTENTS.

### COMMUNICATIONS.

### THE DENTAL REGISTER,

**A MONTHLY MAGAZINE DEVOTED TO THE INTERESTS OF THE**

### DENTAL PROFESSION.

*Terms Reduced to* $2.00 *Per Year. Single Copies,* 25 *Cents.*

### EDITED BY PROF. J. TAFT, D.D.S.

—AND—

### Published by SAM'L A. CROCKER & CO., Ohio Dental and Surgical Depot.

The volume commences in January and closes in December.

The REGISTER being issued monthly is always fresh, therefore Indispensable to the practitioner who desires to keep posted up with the new inventions and improvements which are daily developed. Neither expense nor effort will be spared to give value and variety to its contents. Articles will be illustrated with well executed engravings whenever they will add to the interest or assist to explanation.

Its extensive circulation and frequency of issue make it one of the BEST DENTAL ADVERTISING MEDIUMS in the United States.

All subscriptions must begin with January or July.

Local or special notices, five lines or less, will be inserted for $3 each insertion; over five lines, 50 cts. per line.

All advertising bills payable in advance, or at furthest, after the issue of the first number containing the advertisement. All transient advertisements to be paid for in advance.

☞CONTRIBUTIONS SOLICITED.

Exclusively Editorial Communications should be addressed to the Editor.

The latest number of the REGISTER may be ordered with or without other goods at any time, and all letters should be addressed to

**SAM'L A. CROCKER & CO., Ohio Dental and Surgical Depot, Cincinnati, O.**

